# The Welfare Consequences and Efficacy of Training Pet Dogs with Remote Electronic Training Collars in Comparison to Reward Based Training

**DOI:** 10.1371/journal.pone.0102722

**Published:** 2014-09-03

**Authors:** Jonathan J. Cooper, Nina Cracknell, Jessica Hardiman, Hannah Wright, Daniel Mills

**Affiliations:** Animal Behaviour, Cognition and Welfare Research Group, School of Life Sciences, University of Lincoln, Lincoln, United Kingdom; CNRS (National Center for Scientific Research), France

## Abstract

This study investigated the welfare consequences of training dogs in the field with manually operated electronic devices (e-collars). Following a preliminary study on 9 dogs, 63 pet dogs referred for recall related problems were assigned to one of three Groups: Treatment Group A were trained by industry approved trainers using e-collars; Control Group B trained by the same trainers but without use of e-collars; and Group C trained by members of the Association of Pet Dog Trainers, UK again without e-collar stimulation (n = 21 for each Group). Dogs received two 15 minute training sessions per day for 4–5 days. Training sessions were recorded on video for behavioural analysis. Saliva and urine were collected to assay for cortisol over the training period. During preliminary studies there were negative changes in dogs' behaviour on application of electric stimuli, and elevated cortisol post-stimulation. These dogs had generally experienced high intensity stimuli without pre-warning cues during training. In contrast, in the subsequent larger, controlled study, trainers used lower settings with a pre-warning function and behavioural responses were less marked. Nevertheless, Group A dogs spent significantly more time tense, yawned more often and engaged in less environmental interaction than Group C dogs. There was no difference in urinary corticosteroids between Groups. Salivary cortisol in Group A dogs was not significantly different from that in Group B or Group C, though Group C dogs showed higher measures than Group B throughout sampling. Following training 92% of owners reported improvements in their dog's referred behaviour, and there was no significant difference in reported efficacy across Groups. Owners of dogs trained using e-collars were less confident of applying the training approach demonstrated. These findings suggest that there is no consistent benefit to be gained from e-collar training but greater welfare concerns compared with positive reward based training.

## Introduction

The use of collar mounted electronic training aids, such as radio fence systems to deter roaming, anti-bark devices and manually operated remote training devices is controversial and their use has been banned in some countries, whilst being the focus of considerable political debate in others [1]. For critics of these devices (often called shock collars or, less emotively, e-collars), they represent an unacceptable means of correcting undesirable behaviours [2], whilst others claim they can be useful tools for addressing behavioural problems in pet dogs [3], [4].

The technical features of manually operated e-collar systems has recently been described by Lines et al [5], but broadly speaking they consist of a collar mounted device capable of delivering a short electric stimulus to the neck of a dog via two protruding blunt electrodes. The device is controlled by a hand set, which typically provides a number of settings governing the intensity and duration of stimulus. Most modern devices also allow handler-operated pre-warning cues such as an auditory or vibration signal to precede the electric stimulus. These in combination with other cues, such as verbal commands, offer the potential for avoidance learning by dogs [6] which potentially allows the handler to train more desirable behaviour in a given situation.

The arguments for and against their use have recently been reviewed by the Companion Animal Welfare Council [1], which also highlighted the emotional level of argument used and lack of scientific evidence to draw solid scientific conclusions for welfare-based policy decisions on this matter. The emotion of the argument is reinforced by spectacular public demonstrations of the misuse of these devices on sites like YouTube (e.g. http://www.youtube.com/watch?v=_T9qiGCq5sk, the first video to come up on this site when the term “shock collar” was entered as a search term on this site 19/8/13). There is, however, a lack of description of the immediate responses of animals to the use of these devices in the scientific literature, on which to base scientific and practical considerations. There are some clear theoretical welfare risks, such as the failure to link delivery of the e-collar stimulus with clear conditioning stimuli, or poor timing of response and reinforcement [3], [6], [7], which have been investigated experimentally [8]–[10]. These studies show that these devices have the potential to cause distress and pain, but do not address the question of whether the use of these devices necessarily causes distress; i.e. when used in accordance with best practice by trainers experienced in their use. Indeed it has been suggested that from a theoretical perspective, efficient avoidance conditioning may not always be a significant cause for welfare concern [1].

Although organisations such as the British Veterinary Behaviour Association (formerly Companion Animal Behaviour Therapy Study Group, who advise the veterinary profession in the UK on related policy especially towards pets) state that other reward based methods are similarly effective without the associated welfare risks [11], there do not appear to be any scientific studies to corroborate this statement, especially in relation to efficacy equivalence. Indeed, an experimental study examining the effect of rewards and punishment in the control of “instinctive” behaviour by dogs, concluded that “negative reinforcement and punishment may be desirable and necessary additions to positive reinforcement techniques” [12]. Advocates of such devices suggest they are particularly useful for correcting behaviour at a distance from the operator during off lead activity, such as poor recall, or livestock chasing, when, for example, a food reward cannot be delivered remotely; and in previous studies, these indications were reported to be the two commonest reasons for using such devices in the UK [13], [14].

Studies of dogs undergoing e-collar training have also tended to focus on sub-populations of dogs such as those trained for police work [10], hunting [15] or model populations of laboratory dogs [9]. These populations do not, however, represent the context of their most common use, i.e. with the companion/pet dog population [13]. Furthermore, where studies used older devices, it is possible they are not representative of more modern devices. Retrospective studies, such as Blackwell et al. [13], have been undertaken on pets and found that the use of rewards was associated with a higher rate of success compared to the use of an e-collar for controlling chasing, but, as the authors acknowledge, this may simply reflect differences in severity of the problem between the two sets of respondents. When considering the necessity of a procedure which has the potential to cause harm, it is essential to consider both efficacy and welfare impact of best practice in situ, and to date no study has addressed both of these factors in relation to the use of e-collars in training.

In this study, we aimed to fill three important gaps in our knowledge of the use of e-collars for training pet dogs. Firstly, we described the responses of dogs in the field to training with an e-collar. Secondly, we investigated whether the welfare of dogs trained with an e-collar was necessarily compromised in comparison to approaches which did not rely on use of e-collars, when trying to address the most common problems for which e-collars are often advocated. Finally we investigated the efficacy of e-collar training in addressing these problems in comparison to other approaches. In the first study, which also acted as a preliminary for the main experimental study, we used largely qualitative observational methods to describe the responses of dogs being routinely trained with e-collars, since accurate information from the everyday use of these devices has been missing from the scientific literature [1]. In the main experimental study we used the information gained from this initial work to execute a quantitative assessment of the behavioural and physiological effects of different training regimes on animals exhibiting typical problems for which e-collars are advocated. By controlling for trainer and method of training, we were able to evaluate whether the use of an e-collar produced a significantly different result compared to a regime that did not use an e-collar, both in terms of the welfare of the subject being trained and the resolution of the problem for which the owner was seeking help. This latter study was conducted using e-collar training protocols that were consistent with the published recommendations advocated by collar manufacturers [16]–[19] and delivered by trainers with considerable experience of training with and without e-collars. Data from these dogs were compared with data from dogs trained by the same trainers but without e-collars and by trainers who were members of the APDT (UK), an organisation that does not advocate the use of e-collars. By doing this we could control for the risk of any potential bias towards the use of the e-collar.

### Study Design

The paper presents findings of two studies; a preliminary study involving nine dogs was used to generate initial qualitative data on the use of these devices under typical conditions and refine data collection techniques in the field. This was followed by a larger, controlled study which involved 63 dogs. For this, volunteered subjects who had been referred for problems commonly addressed using e-collars such as recall problems and livestock worrying [13] were allocated with the informed consent of owners to one of three Groups; one using e-collars and two control populations where dogs were not exposed to e-collars (Table S3 in File S2). The e-collar treatment Group (Group A) consisted of dogs referred to professional trainers who were experienced in the use of e-collars to improve off lead recall. Control Group B were dogs referred to the same trainers but trained without the use of e-collars, whilst Control Group C included dogs with similar behavioural problems to those in Group A, but referred to professional trainers who were members of a professional training association focused on reward based training, that do not allow use of e-collars (or other potentially aversive techniques or equipment) by their members (Association of Pet Dog Trainers, UK). Dogs in Groups B and C were subject to the same protocols as those in Group A but with no use of e-collars. Training focussed on improving off lead recall when dogs were exposed to livestock (sheep, poultry) and other dogs. Behavioural and physiological data that related to dog's emotional state [8], [20] were collected during training to assess the immediate impact of exposure to e-collar stimulus in comparison to control Groups, as well as adaptation to training protocols. Dogs were allocated to treatment Group A and control Groups B and C using owner's pre-training assessment of the nature of the referred problem and its severity in order to balance these factors across the three Groups, and owners were surveyed following training to assess the efficacy of training.

## Methods

Ethical Statement: Ethical approval was provided by University of Lincoln Research Ethics Committee following discussion with Home Office Inspectorate in September 2008 for the preliminary study and September 2010 for the main study. Ethical approval was granted as the devices were legal in participating countries and the research team were not modifying trainers' normal use of e-collars. As part of the ethical considerations relating to this project, only adult dogs (over 6 months of age) with no previous experience of e-collars were used, and only subjects that had been voluntarily referred by their owners to trainers who would normally consider the use of e-collars for managing the behavioural problem for which they were referred were enrolled in the study. Owner consent forms were provided to owners prior to the recruitment of their dogs and all the owners of the dogs gave permission for their animals to be used in this study.

### Preliminary Study

A preliminary study was used to generate initial qualitative data on the use of these devices under typical conditions and refine data collection techniques in the field for the subsequent more controlled study. This included: assessing if saliva could be reliably collected in the training context without interfering with the training programme; evaluating the use of video data collection in the field; and developing an ethogram of behavioural responses to training for the main study. Data collection was focussed around the initial exposure to e-collar stimuli, when used to resolve the behavioural problem that was the basis of referral. For this preliminary study, trainer contact details were obtained from publically available marketing (e.g. websites, magazine advertisements) or through collar manufacturers. Nine visits were conducted with four trainers who had 1 dog, 1 dog, 2 dogs and 5 dogs booked for e-collar training respectively; all were willing to allow video recording of the training. 8 dogs had been referred for sheep chasing and 1 for poor recall. Each dog received training over short periods on a single day. Training occurred in rural locations (i.e. farm yards and fields).

One trainer, who was training a single dog for improved recall, followed a protocol that was broadly similar to that advocated by collar manufacturers [16], in that the trainer initially established the intensity of collar setting that caused a mild response in the dog, and used this setting in combination with pre-warning cues to train the dog to return or recall on command. The remaining 3 trainers were training 8 dogs referred for sheep chasing and they adopted a different approach. The collar was fitted prior to exposure to sheep and there was either no assessment of dog's sensitivity to electric stimulation prior to training (two trainers of 3 dogs) or the dogs received a single low intensity stimulation to check the collar was working (1 trainer of 5 dogs). Thereafter, for all but one dog (which was exposed to a setting at the higher end of available range) the trainers selected the highest setting available on the device and dogs were allowed to roam off-lead in a field, where sheep were present. If dogs approached sheep, then the trainer would apply an e-collar stimulus using the high setting with timings of their choice. These trainers stated that they aimed to associate proximity to or orientation towards sheep with the e-stimulus, and consequently did not plan to use pre warning cues such as the collar mounted tone or vibration stimuli as a predictor of electric stimulation.

Saliva was collected at 4 sample periods to allow assay of salivary cortisol [21]–[23]. These were on first arrival at the training location (Sample0), about 15 minutes after the e-collar had been fitted to the dog where it was allowed to engage in moderate exercise, but where no electrical stimuli had been applied (Sample1), about 15 minutes following final exposure to electronic stimulus during training (Sample2), and about 40 minutes following training (Sample3). These timings had been drawn from relevant research into dog's responses to potentially arousing stimuli [24] and verified by the research team [25] in a training context. In this part of the study we did not control for time of day, as we were dependent on availability of trainers, with training sessions normally occurring between 10am and 2pm on each day. However studies of patterns of cortisol secretion in owned dogs rarely find evidence of circadian patterns and any temporal patterns are best described as episodic, relating to key events in the day, rather than light dark cycles [26]–[28]. For this, a large cotton bud was placed towards the back of the dog's mouth, and the saliva extracted before being immediately stored on ice, prior to storage at −40°C. At the end of the preliminary study samples were assayed by Food and Environment Research Agency (FERA) using standard protocols. The sampling technique was simple and effective, involved minimal restraint and could be employed without interfering with training. All dogs readily supplied adequate saliva with cheese used as a lure to stimulate interest and salivation.

Behavioural data were collected by the research team on hand held video cameras before, during and after the exposure to the electric stimulation. Six of the 8 dogs referred for sheep chasing only engaged in one or two approaches, and received a single application of the electric stimulus each time they approached sheep, which led to a cessation of approach. One dog referred for sheep chasing did not approach sheep during the training sessions, but received two stimuli at points when it was orientated towards nearby sheep. One dog received 5 exposures to e-collar stimuli before approaches ceased. As dogs were relatively free to roam open fields during training, video operators chose to position themselves where they could have best view of dogs when in proximity to sheep. As a consequence, it was not possible to have full video records of the entire training period, but good records were made of the period immediately before and immediately after approach to sheep and exposure to electronic stimulation.

For analysis of behaviour before and after exposure to e-collar stimuli, periods of up to 30 seconds prior to and after each exposure based on known times of application were used. Video analysis was conducted by a trained video observer who was independent of the research team in the field and blind to the purpose of the study. The draft ethogram included: time spent in postures such as sit, stand, walk and run; tail position and movement; panting; overall behavioural state including excited, relaxed, tense; and the frequency of number of activities drawn from studies of training in dogs, as well as studies of aversion or anxiety [8], [29]–[31]. These included vocalisations, lip-licking, yawning, paw-lifts and body-shakes. Finally the video observer was asked to note any unusual changes in behaviour during the observations.

As the length of time in view during data collection varied between samples, data for behavioural states and postures were converted into percentage of observation time. These provide useful, independently documented field observations of pet dogs' responses to e-collar use in the field. Descriptive statistics only are presented for these behavioural data. As saliva samples could be sampled consistently, these data were analysed using a repeated measure ANOVA on log transformed cortisol concentrations, with post-ANOVA Tukey test used to identify differences between sample periods.

## Results: Preliminary Study

Video analysis of the preliminary study noted some variation in the immediate reaction of dogs to each application of stimulus, but stimulus reaction could be broadly described as an abrupt change in locomotor activity, normally from walking or running to abrupt halt, or other distinct change in direction of travel and gait. The one exception was the dog trained for recall alone with a warning stimulus and on a lower setting than the sheep chasers, and whilst an apparent response to e-collar stimulus was detected in terms of change in orientation and posture, this appeared less pronounced than that observed in sheep chasers.

Dogs showed a number of additional changes in behaviour in the period following electric stimulus presentation, compared with behaviour prior to stimulus presentation. Dogs showed an increase in vocalisation, with none recorded prior to first stimulus compared to a total of 13 “yelps” and 5 “whines” after exposure. There was a change in tail carriage from principally an elevated carriage prior to exposure (with only 2% of time was the tail between legs) to the tail being between legs 20% of the time following exposure. Prior to stimulus application the dogs were generally described as being in a neutral (40% of time) or investigatory (20%) state with only 10% of time described as tense; whereas afterwards, dogs were tense for 50% of the time and spent only 5% of their time engaged in investigatory behaviour. A small number of yawns and paw lifts were observed after stimuli, but none seen before exposure. Bouts of lip licking and body shaking were recorded before and after exposure at approximately the same rate. Finally there was an increase in owner interaction by the dogs after exposure to the stimuli (56% of time compared with 14% prior to stimuli), with several dogs looking towards or returning to their owners soon after application of stimulus. On returning to owners, dogs received praise and attention.

**Figure 1:**
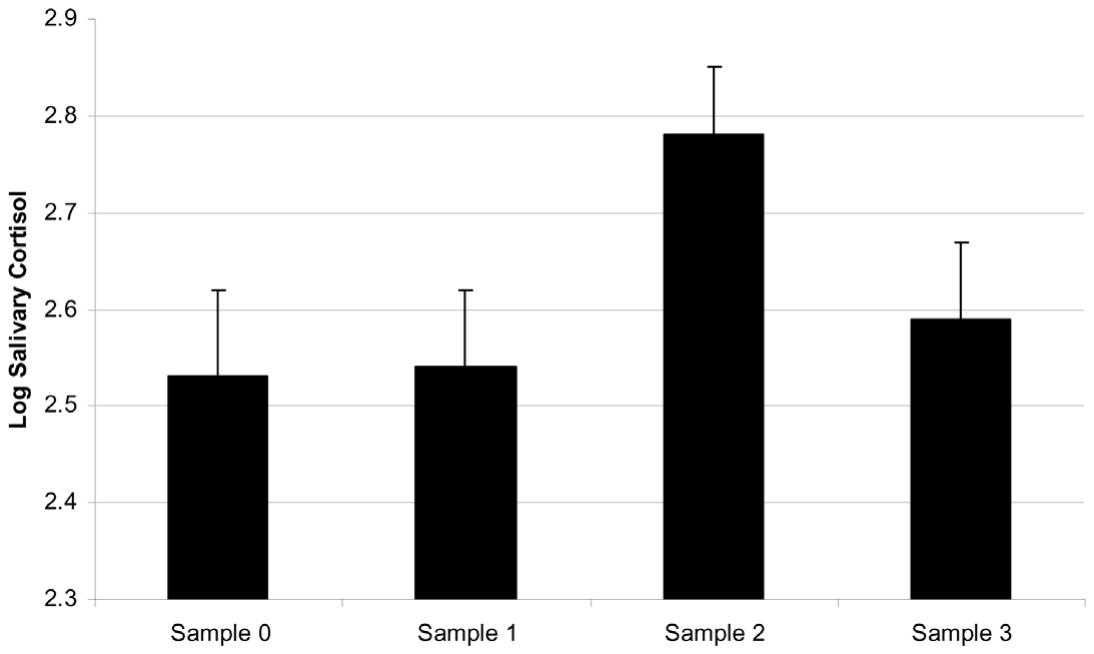
Log_10_ salivary cortisol (mean ± SE) on arrival at training centre (Sample 0), following training without e-collar when dogs were allowed free exercise (Sample 1), 15 minutes following training with an activated e-collar (Sample 2) and 40 minutes following training with e-collars (Sample 3).

There were individual differences between dogs in salivary cortisol output, F_8,23_ = 3.44, p = 0.009, and also sample time effect (F_1,7_ = 3.29, p = 0.041) with post-hoc Tukey test indicating a difference between Sample1 prior to training and Sample2 following exposure to e-collar stimulation (T = 2.89, p = 0.042), suggesting that salivary cortisol following exposure to sheep and training involving e-collar stimuli was elevated in comparison to the pre-training sample (Figure 1).

### Main Study

The study investigated the immediate effects of exposure to e-collars in a pet dog training context, using experienced e-collar trainers (Group A) and compared their responses with a population presenting to the same trainers with similar behaviour problems for training without the use of an e-collar (Group B) and a similar population presented to trainers who do not advocate the use of e-collars in training (Group C). Data collection focused on behavioural and physiological measures of emotional state before, during and after training as well as efficacy. The choice of sample size (21 in each Group) considered the population sizes used in previous between-subject design studies examining the effect of e-collars in more extreme situations (15–16 subjects in Schilder and van der Borg) [10] with an additional 40% to increase sensitivity, given an anticipated smaller effect size. Differences detected at this level, would be substantial enough to be considered practically important, while reducing the risk of Type I errors which might confuse the interpretation of main effects. However, it is recognised that other potentially valuable effects may not be detected as significant using this sample size and so a strategy was developed to accommodate this in statistical analysis and interpretation of results.

Prior to allocation to Groups a questionnaire was used to collect data on the general characteristics of dogs, their past training history and information on why owners were referring dogs for training. Owners were asked to broadly rate the intensity of the main referred problem as; 1 “Always displayed”, 2 “Frequently displayed”, 3 “Occasionally displayed”, 4 “Rarely displayed” and 5 “Never displayed”. Recruited dogs were primarily selected on the basis of attention and recall related problems (including livestock worrying and wildlife chasing) and the need to train a recall task at distance. Reason for referral was the main selection criterion as it was important that the control dogs presented similar behavioural problems and similar levels of severity as those dogs exposed to e-collars. Dogs younger than 6 months of age or with prior experience of electronic training devices were excluded.

Two experienced dog trainers were nominated by The Electronic Collar Manufacturers Association (ECMA) to train dogs in Groups A and B, with equal numbers of dogs allocated to each Group and each trainer working with half the dogs in each Group. The trainers used in Groups A and B commonly used e-collars to address these problems, but did not use these collars exclusively or with every case referred to them. Dogs were allocated to either Group A or B by the research team, based on information provided by owners prior to training on the nature of the referral and severity of problem. The ECMA nominated trainers had no influence on allocation to Group, but if following interview by the research team, owners expressed a preference for or a concern against training with e-collars, they were swapped between Groups with a dog with equivalent training problem and severity. This represented a small number of owners (2 pairs i.e. 4 dogs swapped).

For Control Group C, two trainers with a similar amount of dog-training experience to the trainers used for Groups A and B and who belonged to a professional training organisation (Association of Pet Dog Trainers, UK; APDT, UK) which is opposed to the use of e-collars were recruited to train the same number of dogs presenting with similar problems. Dogs were selected for this Group from volunteers to match dogs studied in Treatment Group A based on reason for referral and severity of problem. Volunteered dogs therefore were allocated to one of three Groups (Table S3 in File S2). The average age of dogs used in the study was 46 months and there was no significant difference in age of dogs between the three Groups. Thirty four out of the sixty three dogs were female (54% of sample), with similar numbers of female dogs in Groups A (n = 13) and C (n = 12), but slightly less in Group B (n = 9), but this difference was not significant (X^2^ = 1.661, df = 2, p = 0.436). Gundogs and cross breeds were the most commonly referred breed-types, represented by 16 dogs each (51% of the sample in total). The remaining dogs were pastoral breeds (n = 11, 17%), terriers (n = 8, 13%), hounds and working breeds (both n = 6, 10% each). There were no representatives of toy or utility breeds as defined by The Kennel Club in the UK (Table S3 in File S2).

There was, therefore, no difference in age profile, sex ratios or breed prevalence between the three Groups. The primary justification for the inclusion of the three Groups used was as follows: any significant differences between Group A versus B and C would most likely reflect the effect of the use of an e-collar in training; whereas differences between Groups A and B versus C would most likely reflect either trainer or environmental effects. As previously mentioned, the inclusion of Group C, ensured that we matched for trainer experience and familiarity with preferred training techniques (including their choice to include or exclude e-collar use). Therefore differences between A and C can be considered to reflect differences between best practice use of the e-collar and best practice which excluded the use of an e-collar. When trying to draw conclusions about the welfare implications of an intervention it is important to triangulate the available evidence in order to make the most robust inferences. Accordingly in the discussion below, we consider the significant effects after correction for false discovery and then evaluate these in light of the more marginal effects (i.e. effects that would have been significant if the difference observed had been replicated in a sample size twice that used).

#### Dog Training protocols

During training, data were collected over a period of up to 5 days covering introduction to e-collar and other training stimuli and the period of initial modification of behaviour. For Group A the choice of collars and precise training regime were determined by the trainers, using e-collars with a variable setting to allow the operator the opportunity to determine the level at which the e-collar stimulus was to be delivered, and a pre-warning cue which might allow dogs over time to modify behaviour prior to exposure to e-collar stimulus. Trainers only worked with their preferred make and model of device, which were Sportdog SD-1825E (n = 11) and Dogtra 1210 NCP (n = 10). E-collars were chosen that had both tone and vibration pre-warning cues, however, with the agreement of the trainers, only vibration cues were used during training to ensure video analysis was blind to treatment.

Dogs' individual training regime was determined by the trainer and followed typical good practice for resolving the problem under referral given the chosen method. Dogs in Group A were to have the working level of e-stimulus determined on day 1 of training, whilst on subsequent days non-compliance with trainer given cues would be associated with potential exposure to the e-stimulus, with the pre-warning stimulus used as desired by the trainer. Dogs in this Group were also exposed to positive reinforcement such as food, play and/or praise for compliance. Dogs in control Groups B and C wore a dummy collar (de-activated e-collar) to control for collar wearing and ensure observers of video tapes were blind to treatment. On the final training day (normally day 5), all dog owners conducted training under instruction from the trainers. For a small number of dogs, where trainers felt training had progressed sufficiently, this final owner training day was day 4, and the dogs did not return for a 5^th^ day of training. This represented one dog from Group A and one dog from Group B.

Dogs were trained at one of two training centres. Dogs in Groups A and B were trained at a farm location near to Edinburgh during Autumn-Winter 2010. Dog training initially occurred in a field setting with a small flock of sheep and small flock of poultry penned in the training field. When weather conditions were not conducive to outdoor training, the training was relocated to a yard on the same farm with similarly penned animals. Dogs in Control Group C were trained at Riseholme near Lincoln in Spring 2011, with a field set up to replicate conditions originally used in the Edinburgh training centre. The timing of data collection was related to the availability of professional trainers, and the consequences of this will be discussed in light of findings of the study. Each training session lasted approximately 15 minutes and each dog received two training sessions per day, one in the morning and one in the afternoon. Behavioural data were collected by video recording for the full duration of each training session, on days 1, 2, 3, 4 and 5 as applicable.

#### Behavioural Data - Video Analysis

An ethogram based on review of the preliminary study, and with input from a related study on long term effects of e-collar training [14], was developed to cover time spent in different postures, in different qualitative behavioural states, tail positions and panting and the frequency of activities (Tables S1 and S2 in File S1). Video analysis was conducted by four observers with experience of behavioural recording who were blind to Groups and the objectives of the study. Each observer received training to become familiar with the ethogram developed for this study and the data collection protocols, and to allow assessment of inter-observer reliability. Inter-observer reliability was tested by allocating four videos to different observers at an early stage of analysis. Consistency in scoring was assessed by calculating the correlation co-efficient r for the behavioural categories. Where r>0.8, it was assumed there was good agreement between observers' scores and they were reliably following the sampling method. Where there was poor agreement (r<0.8), observers received further training to address inconsistencies. This was only necessary for one observer, who following retraining and re-analysis of early tapes was in good agreement with all other observers for the rest of data collection. Training videos were allocated so that each observer had similar numbers of dogs from each Group, although they were also blind to this partition.

Data from training videos were extracted from video tapes using a Microsoft Excel based check-sheet with each video having two sets of observations recorded. The first observation used an instantaneous scan sample technique where videos were sampled once per minute (up to 15 scans per video). At each sampling point the dog's posture (sit, stand, walk, run), overall behavioural state (relaxed, tense, excited, neutral), distance to trainer and distance to owner, tail carriage and movement, and panting were recorded. If dogs were out of sight or behaviour could not be determined at the sampling point then each category of behaviour was recorded as unknown. The second observation consisted of a continuous sample of the frequencies of key behavioural events. These included oral activities (yawn, lip licks (with or without food)) and vocalisations. In addition, any time out of view was recorded. This allowed calculation of the frequency of events per minute of time in view for analysis. Categories used in these ethograms were derived from previous studies investigating anxiety and arousal in dogs [8], [29]–[31] as well as the experience of data collection during the preliminary study and project AW1402 [14].

Efficacy of training was assessed by questionnaire distributed to owners one week following training. Where owners did not return this questionnaire, the questionnaire was resent. This resulted in responses from all 21 owners whose dogs joined Groups A and C, and 19 returned questionnaires for Group B. Questions related to the owner's perception of improvement in both their dog's behaviour, whether they were continuing to use the training techniques they had learnt during the sessions, and their confidence in using these techniques. Responses were scored using a five point semantic differential scale for each item, for example from very confident to not confident, or from very satisfied to very dissatisfied, which were then allocated numerical scores from 1 to 5 for analysis.

#### Statistical Analysis

Data analysis was completed in Minitab 15.0 using parametric approaches where appropriate on raw data or following transformation. Rare behaviours seen in less than 10% of dogs were removed from analysis, as were distance to owner and distance to trainer as these could not be reliably assessed for many videos as human subjects were out of frame for long periods. Where data were collected over several phases of study, then a repeated measure design was conducted with dogs nested within Group used as the between subject variable, or where data did not meet requirements of parametric analysis sampling period effects were assessed using Friedman ANOVA on each Group.

This approach, however, resulted in some loss of dogs from analysis where data were not recorded over all sampling periods. For example where dogs ceased training on day 4, but more particularly with sampling of urine where some owners (n = 23) were not able to extract first passage urine from their dogs on every training day. As no sampling order effects were found during preliminary analysis, the data for each dog were averaged across sampling periods in order to provide data on every dog in each Group. These were analysed with a one way ANOVA for parametric data or Kruskal-Wallis test for non-parametric data. A post-hoc Tukey test was employed to test for differences between Groups where Group effects were identified from ANOVAs (or pair wise Mann-Whitney for non-parametric data). Finally for dogs in Group A, although it was not possible to determine the number of applications of electronic stimulus during training, data were available for the device setting during each training session, which allowed analysis of co-variance between behavioural responses and collar settings (controlling for trainer/collar brand) for parametric data and Spearman rank correlation for non-parametric data.

As the behavioural data analysis included multiple comparisons of related data, correction factors were used to control for Type I errors. For this the False Discovery Rate method developed by Benjamini & Hochberg [32], [33] was used to take into account the analysis of a large number of behavioural measures. Variables that met these corrected criteria are presented in bold in Tables S4 and S5 in File S2 and described in text as being a significant effect.

## Results: Experimental Study

### Reasons for Referral in Sample Population

The majority of dogs referred had chasing or worrying as their owner's primary concern (51 dogs or 81% of sample), involving chasing sheep/lambs, horses, rabbits, joggers and cars, or a combination of these. These were similarly represented in the three Groups. Nine dogs (14%) were referred for general recall problems without the owners reporting any issues with chasing or worrying, whilst three dogs (5%) had owners whose primary concern was aggressive encounters with other dogs whilst off lead (Table S3 in File S2). The majority of owners rated the problems as either 1 (“always displayed”, 31 dogs or 49% of sample) or 2 (“frequently displayed”, 24 dogs; 38%). Six dogs were rated as occasionally displaying the problem, two as rarely, but the owners had wanted further advice on addressing off-lead problems. A numerically higher proportion of dogs in Group C were described as always showing the referred problem (67% of Group) compared with 48% of Group A and 33% of Group B, but this difference was not statistically significant (X^2^ = 4.79, df = 2, p = 0.091).

### Behavioural Measures During Training

There were no day effects on dog activity, panting, behavioural state or tail carriage over the five training days. Dogs in Groups A and B were recorded as spending roughly half of their time walking during training, which was significantly more than dogs in Group C, who were observed significantly more often to be standing during the training sessions (Table S4 in File S2). There were also significant differences between Groups in sitting which was most common in Group A and least common in Group C. No differences were found in tail carriage or movement between Groups.

Panting appeared to be twice as common in Group A dogs (20% of scans) as Groups B and C (both about 10%), however, this was not a significant effect. Close examination of the data indicated that a small number of dogs in Group A showed elevated rates of panting; 4 dogs were panting in over 50% of scans, compared with none in Groups B and C. There was no evidence of a difference in percentage of scans in the behavioural states relaxed, ambiguous or excited (Table S4 in File S2) between the three Groups. There was a difference in time spent in a tense state, as dogs in Group C spent less time tense than dogs in Group A (Tukey, t = 3.14, p = 0.007), but no difference between Groups A and B or B and C (t<1.87, p>0.16).

There were no day effects on continuous recorded activities. There were differences between the Groups in the rates of a number of activities (Table S5 in File S2) Overall, lip-licking was similar between the three training approaches, however, when this was separated between lip-licking in association with food, then Group C dogs showed more food related lip-licking than dogs in either Group A or B. In contrast, differences in lip licking in absence of food were not significant at the sample sizes in this study.

**Figure 2:**
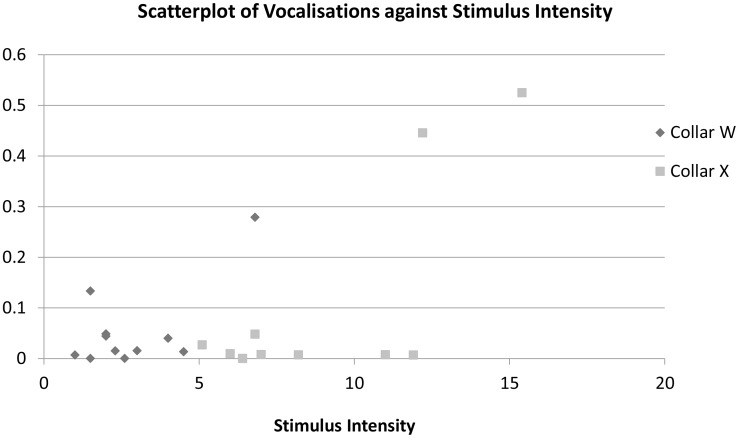
Scatter plot of rate of vocalisations per minute against average collar settings (Stimulus intensity) used during training for the two collars (W being Sportdog SD-1825E (n = 11) and X being Dogtra 1210 NCP (n = 10)) used with Group A dogs.

Dogs from Group A showed more yawning than dogs in Group C (Table S5 in File S2). Sudden movements away from trainer, including rapid turning away of head or body movements, appeared to be least common in Group C, though this was not significant at the sample size of the study. Dogs in Group A appeared to engage in most yelping, though yelping was rare in all Groups and most dogs were not recorded yelping in any training session. It appeared to be about 5 times more common in Group A than in either Group B or C, but this apparent difference was not significant. As with panting, yelps appeared to be primarily observed in a small number of dogs in Group A; the majority of dogs in that Group showed no yelping. There was, however, evidence of a relationship between vocalisations and collar settings for Group A dogs, with yelping (F1,17 = 7.58, p = 0.014) and all vocalisations (F1,17 = 10.7, p = 0.004) increasing with average collar stimulus intensity setting across training days. These differences appear to largely relate to a small number of dogs trained at higher settings showing high frequencies of vocalisations with most dogs in Group A showing no or few vocalisations during training sessions (Figure 2).

Two further aspects of training were found to differ between Group C and both Groups A and B. These were the number of commands given, where dogs in Groups A and B appeared to receive about twice as many commands per training sessions than dogs in Group C (Table S5 in File S2) and sniffing or environmental interactions, which occurred at about half the rate in Groups A and B, then in dogs in Group C.

### Physiological Measures During Training

Overall there were no consistent differences between sampling periods in salivary cortisol, and no evidence of interaction between sampling period and Group, but there was a Group effect on salivary cortisol (F_2, 59,_ = 6.11, p = 0.004), with dogs in Group C (logCort = 3.10±0.016) having higher levels during the study than those in Group B (logCort = 2.92±0.022; LSD, p = 0.001). Values from Group A (logCort = 3.02±0.023) did not differ from those of Group B (LSD, p = 0.08) or Group C (LSD, p = 0.066). These Group differences were found in both the pre-training samples on day 1 and day 5 (F_2, 59,_ = 3.35, p = 0.042) and the post training samples on days 1 through to 5 of training (F_2, 59,_ = 5.32, p = 0.008). Furthermore, when the average pre-training sample measures were subtracted from average post-training sample measures, there was neither an overall difference (Paired t-test, n = 62, t = 0.18, p = 0.85) nor a Group effect (F_2, 59_ = 0.03, p = 0.96).

Overall there was no significant difference in urinary cortisol to creatinine ratios between Groups before (F_2,59_ = 0.91, p = 0.41) or after training (F_2,59_ = 0.03, p = 0.97) with average values of 1.65±0.11 for Group A, 1.69±0.23 for Group B, and 1.64±0.14 for Group C in the four samples taken after training sessions had been experienced. There were also no changes in concentration ratios over the five days of training for any Group. There was no effect of collar setting on any physiological measures in Group A.

### Owner Perception of Efficacy

**Figure 3:**
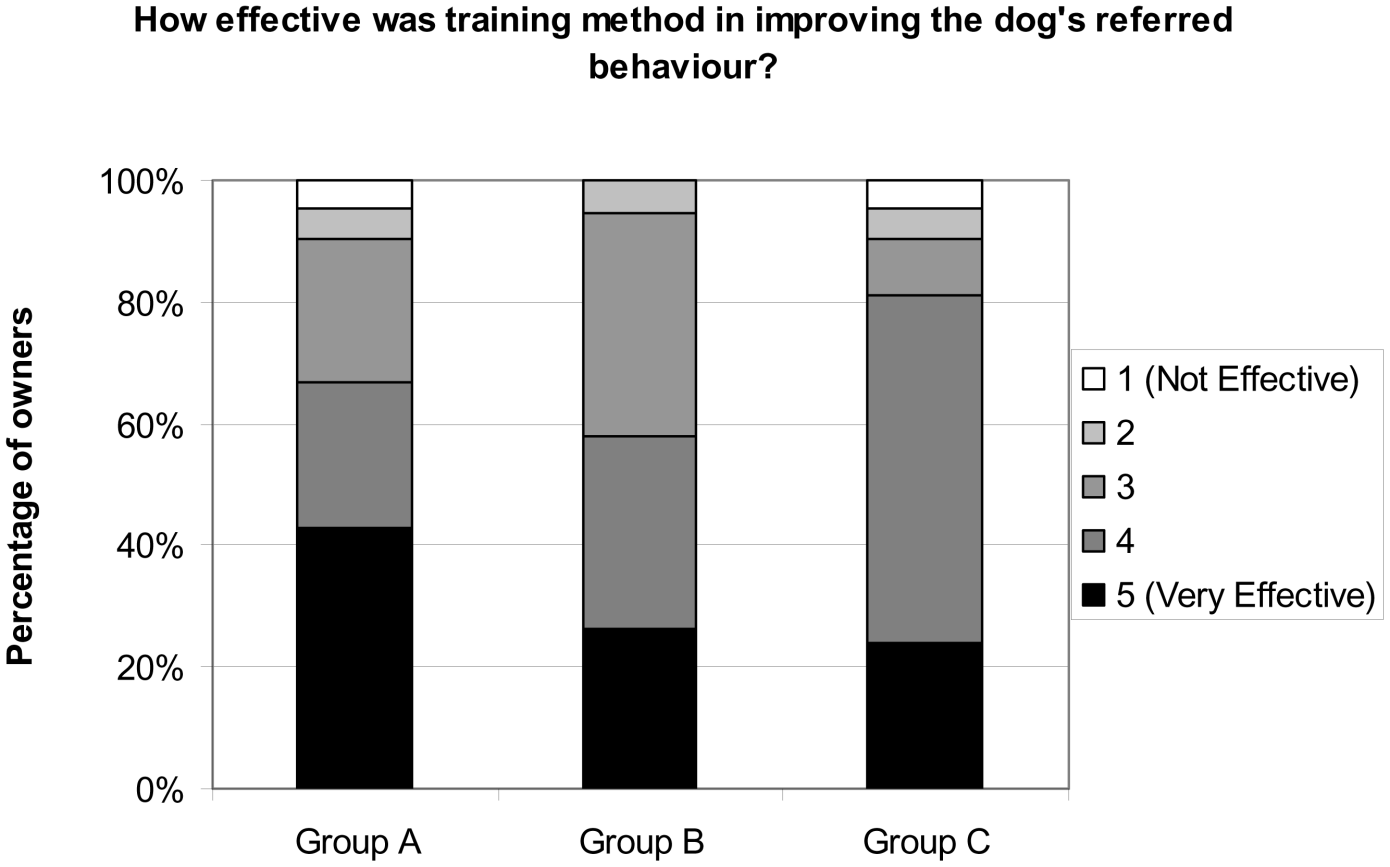
Percentage of owners in each response category indicating that training was effective at improving dog's referred behaviour.

**Figure 4:**
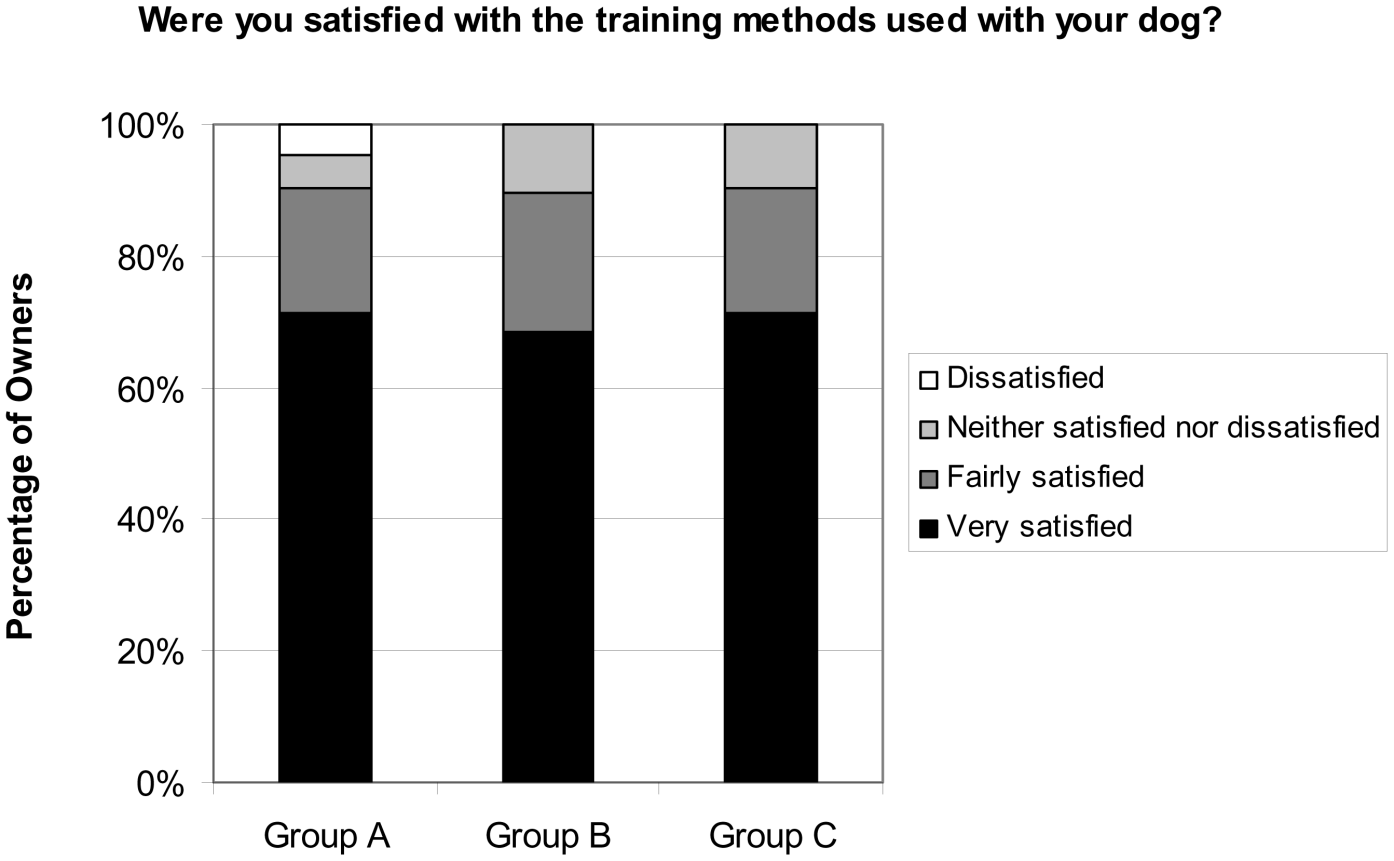
Percentage of owners in each response category who were satisfied with the training methods used.

**Figure 5:**
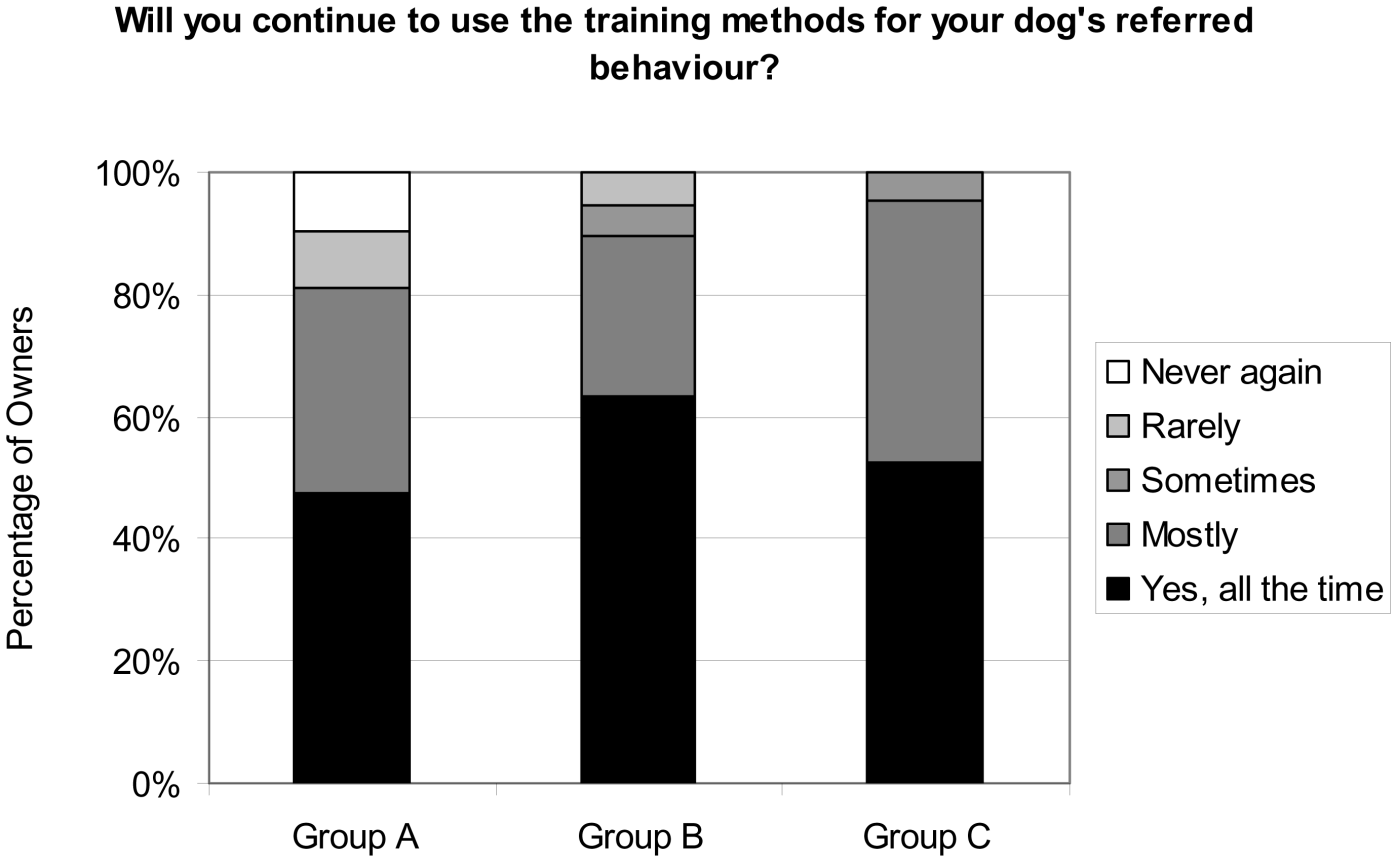
Percentage of owners in each response category who would continue to use the training methods to address the referred behaviour.

Overall, owners were generally satisfied with the training programmes in which they had participated. 88.5% of owners reported they had seen an improvement in their dog's general behaviour and 91.8% reported that there had been an improvement in the obedience problem for which their dog had been referred (Figure 3). There were no significant differences in the responses of owners from the 3 Groups. 18 out of 19 owners (94.7%) from Group B reporting improvement in both measures, whereas 18 out of 21 owners (88.5%) of owners who had participated in both Groups A and Group C, considered that their dog's general behaviour had improved. 19 out of 21 owners (90.5%) from both Groups A and C also considered obedience with respect to the referred behaviour had improved. 90.2% of owners reported they were satisfied with the training advice they received (Figure 4) and 88.5% indicated that they were continuing to use the trainers' advice both for general dog behaviour and in relation to the problem that was the reason for referral (Figure 5). There was no evidence of differences between the three training Groups in these measures of satisfaction.

**Figure 6:**
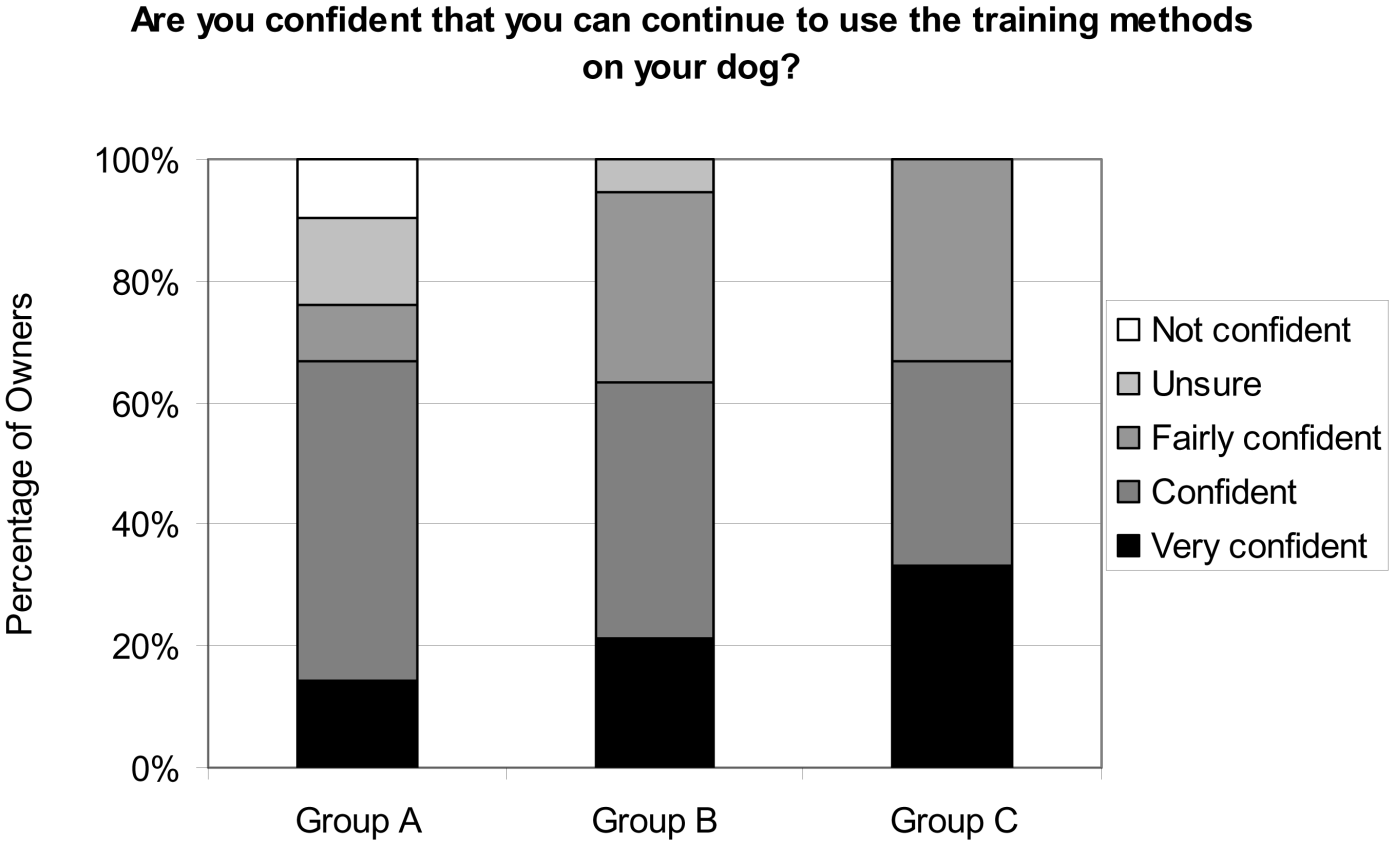
Percentage of owners in each response category who reported they were confident of continuing to use the training methods.

The majority of owners (91.8%) reported they were confident of being able to continue to apply the training techniques. All 21 owners (100%) from Group C, and 18 out of 19 respondents (94.7%) from Group B stated they were confident of continuing to effectively use the training programme, compared with only 16 out of the 21 owners (76.2%) in Group A (Figure 6). Chi squared analysis suggests there was a significant differences in confidence between these three Groups (X^2^ = 8.33, df = 2, p = 0.016), though the size of each of the non-confident cells was small. Investigation using a Fisher's exact test indicated that there was a difference in confidence with training approach between Group A and Group C (p = 0.048) and between Group A versus Groups B and C combined (p = 0.015), whereas no other combinations were significant, suggesting that owners of dogs who experienced e-collar training (Group A) were less confident in applying the training approaches seen than those whose dogs were not trained with an e-collar.

## Discussion

In the preliminary study, only 1 dog trained for improved recall experienced an approach that was similar to that advocated by collar manufacturers in the UK [16], where the dog's sensitivity to e-collar stimulus was assessed prior to training, and where, during training, this level of stimulation was associated with a pre-warning cue or conditioning stimulus. Under these conditions, the trainer (and dog) had the potential to gain greater control over the situation, since higher order conditioning can be used to build an association between the conditioned stimulus (pre-warning cue) and a verbal command to interrupt ongoing behaviour. Although the application of stimulus was discernable in this dog, its response was mild in comparison to the other dogs observed in the preliminary study.

In contrast, trainers aimed to develop an association between the electric stimulus alone and proximity to sheep in the 8 other cases. The development of an aversion response in this way has also been studied in hunting dogs exposed to stuffed or frozen kiwi or kiwi feathers [15], where dogs showed long term aversion to these models (though the study does not present evidence of efficacy with live kiwis). Furthermore, whilst the authors considered the welfare implications of the aversion based training, they did not record the response of these dogs to the electrical stimulus or other measures of the welfare impact on the dog of this experience. This approach to controlling behaviour around prey species requires good timing on the part of the handler, as poor association between the stimulus and related cues has been found not only to be ineffective in changing behaviour [34], but also to result in prolonged elevation of corticosteroids [9].

In our preliminary study, we observed distinct changes in behaviour, including sudden changes in posture, tail position and vocalisations that are consistent with pain and/or aversion in dogs [8], [10]. The significant elevation in salivary cortisol recorded in these dogs after e-collar training, may be due to the e-collar stimulation, and/or the arousal resulting from exposure to prey stimuli in the form of sheep and/or associated chase behaviour prior to stimulation. Nonetheless, the elevation is comparable to those found by Beerda et al [8], and Schalke et al [9] when dogs were exposed to e-collar stimulation without exposure to a potential prey species. Taken together, these results are consistent with exposure to a significant short term stressor in the form of an aversive and probably painful stimulus during training.

The aim of this second study was to assess the efficacy and welfare implications of best practice with respect to a behaviour modification programme including the use of e-collars versus best practice for the same problem while excluding their use. The rationale was that if, under these conditions, we could bring scientific evidence to the discussion of the costs and benefits of these devices in society. In contrast to the field observations of the preliminary study, in this experimental study the trainers using e-collars were observed consistently to undertake an assessment of the dog's sensitivity to e-collar stimulus. Furthermore, a pre-warning cue was paired with exposure to e-stimulus as a conditional stimulus with the aim of allowing dog's to learn to avoid the e-stimulus. Although this “idealised” use of e-collars may represent the way some dogs are trained, it does not represent the methods used for all dogs, as evidenced by our preliminary study.

Trainers of Groups A and B used more commands than those in Group C and encouraged sitting and walking rather than standing. Dogs in Groups A and B also spent less time sniffing and engaging in environmental interactions during training. There was also some evidence (Table S4 in File S2) that dogs in Group B, and particularly Group A spent more time with a lower tail carriage than those in Group C, as well as performing more sudden movements away from the trainer. These results are most parsimoniously explained by differences in training approach since it is unclear how these differences could be consistently associated with the geographical differences between the two training sites or the time of year of data collection. Lower tail carriage is often associated with stress [20], and sniffing might be a displacement behaviour associated with anxiety [10], or may be associated with the use of food rewards by the trainers in Group C, or their willingness to allow dogs to engage in more environmental interactions during training. These trainer based differences would be worth further investigation, to examine if they are simply individual differences, or reflect a more general difference in style associated with training philosophy, since trainers of Groups A and B were recommended by ECMA, and the trainers of Group C were assessed members of the APDT, UK. However, no conclusions should be drawn at this time given that only 4 trainers were observed out of a much larger population who may vary considerably in their interpretation and application of different training approaches

When considering the welfare implications of the inclusion of the e-collar in training, there were significant differences between Groups A and C. Specifically, dogs in Group A were more frequently described as tense and yawned more. Yawning has been identified as a behavioural sign of conflict or mild stress in a number of studies (e.g. 8, [35]). Other marginal differences support the inference that some dogs in Group A were experiencing welfare compromises during training including the incidence of panting and yelping. Closer inspection of the data revealed that the higher levels of yelping and panting in Group A appeared to arise from a small number of dogs. Yelping may be interpreted as a response to pain and was reported as such in Schilder and van der Borg's study [10] and the preliminary study presented above, where dogs were exposed to higher intensity e-collar stimuli. However most dogs in Group A yelped at a much lower rate than reported in the above studies, equivalent to roughly half a yelp per fifteen minute training session, during which time dogs could have received several e-stimuli per session. In Group A, the highest frequencies of vocalisations were associated with the highest settings used on each of the designs of collar.

Panting is normally associated with thermo-regulation in dogs, but appeared to be rarer in the dogs trained in the warmer spring collection period. Panting has also been associated with acute stress in dogs [35] and again there was some evidence to suggest that a sub-population in Group A engaged in most panting during training. These were no clear associations between this behaviour and activity level or collar setting, so it is not possible in the current sample to establish if these dogs were panting as a consequence of the training programme. Finally there was some evidence of more whining in Group C dogs. This vocalisation has been associated with social solicitation [36], attention seeking and/or food begging behaviour [37] in dogs.

There was no significant difference between the three Groups in cortisol levels measured in the medium (urinary) term. However dogs from Group C consistently showed elevated salivary cortisol compared with dogs in Group B, with Group A dogs at an intermediate level but closer to measures of Group B. These differences were found in both the pre-training and post-training samples which suggest that the findings do not relate to the use of e-collars in training protocol. Whilst elevated cortisol can be interpreted as evidence of distress in response to environmental challenges, this is not a uni-valent state, as high arousal associated with positive emotional states can also elevate cortisol as well as there being associations with the level of physical activity [23]. It is therefore important to evaluate differences in cortisol in light of other measures of environmental response such as behaviour. In the preliminary study, the elevated cortisol found post training in the preliminary study is consistent with the negative behavioural responses observed and an interpretation of pain or aversion during training [8], [10], (though as discussed we cannot without potentially unethical controls rule out the potential of enhanced arousal related to exercise and exposure to sheep alone). In the second study, it is harder to explain the differences in cortisol as the behavioural measures were consistent with a negative (albeit less severe) response to stimuli experienced by treatment Group A. Furthermore there was no evidence of differences in cortisol levels between pre-training and post-training samples for any Group. Overall the physiological data from the main study suggest two things: firstly that once the dogs entered training, none of the treatments resulted in large increases in cortisol secretion and by inference arousal or stress; and secondly the differences in salivary cortisol between treatment Groups appear to represent some underlying difference in arousal, perhaps related to time of year, rather than a difference in arousal due to the training programmes.

A common claim by advocates of the use of e-collars is that they are the most effective way to reliably reduce some potentially dangerous behavioural problems, in particular failure to recall or worrying other animals including livestock and other dogs when off lead. Indeed off lead problems have been found to be the most common reasons for using manually operated devices in the UK [13], [14]. For this reason we controlled for reason for referral (behavioural problem) and owner assessment of severity in allocating dogs to Groups, and we conducted follow up questionnaires to assess owner's satisfaction with the training programme and improvements in dog's referred behaviour. The treatment Group and two control Groups were well balanced in terms of reason for referral, with no significant difference between Groups in reason for referral or owner assessment of severity. Owners were generally satisfied with the advice they received from trainers, and on the whole saw improvements in both the referred problem and their dog's general behaviour. Whilst there is the potential for bias in the owners reporting of behaviour, there is no reason to anticipate that this would differ between the three Groups and findings such as these are entirely consistent with owners having the opportunity to work closely with experienced professional trainers over several training sessions. Apart from their being some evidence that Group C owners were more confident of applying the approaches they had been shown, there were no differences in owner satisfaction between the training programmes, or in dog's improvement in behaviour. This suggests that the use of e-collars is no more effective than the use of mainly reward based training to improve off lead obedience.

## Conclusions

Our results indicate that the immediate effects of training with an e-collar give rise to behavioural signs of distress in pet dogs, particularly when used at high settings. Furthermore, whilst best practice as advocated by collar manufacturers mediates the behavioural and physiological indicators of poor welfare detected in the preliminary study, there are still behavioural differences that are consistent with a more negative experience for dogs trained with e-collars, although there was no evidence of physiological disturbance. E-collar training did not result in a substantially superior response to training in comparison to similarly experienced trainers who do not use e-collars to improve recall and control chasing behaviour. Accordingly, it seems that the routine use of e-collars even in accordance with best practice (as suggested by collar manufacturers) presents a risk to the well-being of pet dogs. The scale of this risk would be expected to be increased when practice falls outside of this ideal.

## Supporting Information

File S1 Table S1, Ethogram of behavioural categories sampled by fixed interval scan sampling. Table S2, Ethogram of behavioural categories counted by continuous behavioural sampling.
(DOC)

File S2 Table S3, Treatment Groups in Main Study. These include the numbers of dogs belonging to UK Kennel Club breed types, gender, age, reasons for referral and owner's assessment of severity of referred behaviour. Table S4, Mean (SE) percentage of scans in posture/activity, panting, behavioural state, tail movement and position. F-statistic and p value from one way ANOVA. Group differences identified by post-hoc Tukey t-test; a and b indicate that there are significant differences between groups. Where data did not conform to requirements of parametric analysis, a Kruskall-Wallis test was applied followed by Mann-Whitney test to identify group differences. These measures are marked with an asterisk*. To correct for Type I errors due to multiple comparisons, the False Discovery Rate control (Benjamini & Hochberg 1995, 2000) was applied. Variables in **bold** showed significant effects based on this adjusted criteria. To correct for Type I errors due to multiple comparisons, the False Discovery Rate control (Benjamini & Hochberg 1995, 2000) was applied. To take into account Type II errors, power tests were applied to the sampled data. Variables in *italics* did not meet the False Discovery Rate criteria but application of power tests, suggest that if the pattern of group variation had been found in a sample size approximately twice that of this study (n = 120), then the data would also have met this criteria. Table S5, Frequencies of activities presented as mean counts (SE) events per training session. F-statistic and p value from one way ANOVA. Group differences identified by post-hoc Tukey t-test; a and b indicate that there are significant differences between groups. Where data did not conform to requirements of parametric analysis, a Kruskall-Wallis test was applied followed by Mann-Whitney test to identify group differences. These measures are marked with an asterisk*. To correct for Type I errors due to multiple comparisons, the False Discovery Rate control (Benjamini & Hochberg 1995, 2000) was applied. Variables in **bold** showed significant effects based on this adjusted criteria. To take into account Type II errors, power tests were applied to the sampled data. Variables in *italics* did not meet the False Discovery Rate criteria but application of power tests, suggest that if the pattern of group variation had been found in a sample size approximately twice that of this study (n = 120), then the data would also have met this criteria.
(DOC)
