# Correction: The Welfare Consequences and Efficacy of Training Pet Dogs with Remote Electronic Training Collars in Comparison to Reward Based Training

**DOI:** 10.1371/journal.pone.0110931

**Published:** 2014-10-10

**Authors:** The PLOS ONE Staff

There are errors in the Funding section. The correct funding information is as follows: This is a new analysis of data from a Defra-funded research project (AW1402a: Studies to assess the effect of pet training aids, specifically remote static pulse systems, on the welfare of domestic dogs; field study of dogs in training). The research project was quality assured and independently peer reviewed. University of Lincoln provided financial support for the additional analysis presented in this paper.
