# Supplementary material for: The Welfare Consequences and Efficacy of Training Pet Dogs with Remote Electronic Training Collars in Comparison to Reward Based Training
Source: PLoS One. 2014 Sep 3;9(9):e102722. doi: 10.1371/journal.pone.0102722 (PMC4153538; doi:10.1371/journal.pone.0102722)
Supplement: File S1 — Table S1, Ethogram of behavioural categories sampled by fixed interval scan sampling. Table S2, Ethogram of behavioural categories counted by continuous behavioural sampling. (DOC) [file pone.0102722.s001.doc]

Supporting Information 1. Ethograms

Table S1. Ethogram for Scan Sampled Behaviour

Behaviour Description

| Down | Body is touching the ground |
| --- | --- |
| Run | Fast movement |
| Sit | Dogs bottom is on the floor in a sit position |
| Stand | All four feet are on the ground and fully extended, bottom is in the air |
| Walk | Slow movement |
| Posture Unknown | Dog is not visible or too far away for behaviour to be interpreted. |
|  |  |
| Panting | Mouth open wide, breathing vigorously |
|  |  |
| Tense | Dogs shows a combination of: tense facial posture (muzzle tight), ears back or down, tail held stiffly or between legs, lip licking, rapid jerky head movement |
| Relaxed | Dog shows slow, relaxed movement with no tension in face or muzzle |
| Excited | Dogs shows a combination of: rapid or jerky movement, jumping, panting, ears forward, tail high or wagging, play signals |
| Anticipatory | Dog shows intense focus on a person or task, for example waiting for a command, with ears forward and direct gaze |
| Ambiguous | Dog clearly displays a mixture of ‘tense’ and ‘relaxed’ or ‘excited’ behavioural signs |
| Unknown | Dog is not visible, video recording is unclear, or the behaviours of the dog cannot be clearly interpreted |
|  |  |
| Next to owner: | Dog is positioned adjacent to the owner |
| Close to owner: | Dog is within arms distance (less than 1m) from owner |
| Inter to owner: | Dog is within 1-5 metres from owner |
| Far from owner: | Dog is over 5 metres from owner |
| Unknown to owner: | Dog is not visible or too far away for behaviour to be interpreted. |
|  |  |
| Next to trainer: | Dog is positioned adjacent to the trainer |
| Close to trainer: | Dog is within arms distance (less than 1m) from trainer |
| Inter to trainer: | Dog is within 1-5 metres from trainer |
| Far from trainer: | Dog is over 5 metres from trainer |
| Unknown to trainer: | Dog is not visible or too far away for behaviour to be interpreted. |
|  |  |
| Tail Still: | Tail is held stationary |
| Tail Wag: | Tail moving from side to side |
| Tail Unknown | Dog is not visible or too far away for behaviour to be interpreted. |
| Tail High | Tail is held stiffly and upright, either curled over the back or straight |
| Tail Neutral | Tail is held in the normal carriage position for dog |
| Tail Low | Tail is held down either straight or slightly curled under the dogs legs |

Table S2. Ethogram for Continuous Sampling

| Body Shake | Vigorous movement of whole body side to side |
| --- | --- |
|  |  |
| Elimination | Expelling of faeces or urine |
|  |  |
| Flinch or back away | Generally a quick action where dog lowers body towards floor, (bottom is usually higher than head and dog’s head can generally be turned away), or where dog briefly backs away from owner or collar. Muzzle is often tense and ear position is backwards. Tail can be positioned between legs or downwards. |
|  |  |
| Move or Turn away | Dog turns away from owner or researcher involving either head movement alone or whole body movement. |
|  |  |
| Attention Seek | Any attempt to elicit attention from owner or researcher physically, e.g. jumping up at, pawing, touching with nose. |
|  |  |
| Sniff or Manipulate environment | Dog places nose in close proximity to the ground, air inhalation (sniffing) or snorting can sometimes be heard, and/or uses mouth or a paw to engage with immediate environment e.g. tugging lead, digging ground, picking up sticks or stones |
|  |  |
| Paw lift | One fore limb only is lifted off the ground. It is not directed at any person or object and all other limbs remain on the ground. |
|  |  |
| Groom or Scratch | Dog places mouth on its body or brings part of its body (usually front limb) to mouth and proceeds to lick or nibble at coat or skin. |
|  |  |
| Lip lick | Tongue leaves and re-enters mouth, either following the ingestion of food or when no food has been given. |
|  |  |
| Vocalisation: |  |
| Bark | Short duration, medium pitch, often repeated vocalisation |
| Whine | Long duration, high pitch vocalisation |
| Yelp | Short duration, load, high pitched vocalisation |
| Growl | Medium duration low pitched vocalisation |
|  |  |
| Yawn | Mouth opened wide briefly then shut. |
|  |  |
| Command Given | Owner gives dog a command such as Sit, Down, Stay, No |
