# Supplementary material for: The Welfare Consequences and Efficacy of Training Pet Dogs with Remote Electronic Training Collars in Comparison to Reward Based Training
Source: PLoS One. 2014 Sep 3;9(9):e102722. doi: 10.1371/journal.pone.0102722 (PMC4153538; doi:10.1371/journal.pone.0102722)
Supplement: File S2 — Table S3, Treatment Groups in Main Study. These include the numbers of dogs belonging to UK Kennel Club breed types, gender, age, reasons for referral and owner's assessment of severity of referred behaviour. Table S4, Mean (SE) percentage of scans in posture/activity, panting, behavioural state, tail movement and position. F-statistic and p value from one way ANOVA. Group differences identified by post-hoc Tukey t-test; a and b indicate that there are significant differences between groups. Where data did not conform to requirements of parametric analysis, a Kruskall-Wallis test was applied followed by Mann-Whitney test to identify group differences. These measures are marked with an asterisk*. To correct for Type I errors due to multiple comparisons, the False Discovery Rate control (Benjamini & Hochberg 1995, 2000) was applied. Variables in bold showed significant effects based on this adjusted criteria. To correct for Type I errors due to multiple comparisons, the False Discovery Rate control (Benjamini & Hochberg 1995, 2000) was applied. To take into account Type II errors, power tests were applied to the sampled data. Variables in italics did not meet the False Discovery Rate criteria but application of power tests, suggest that if the pattern of group variation had been found in a sample size approximately twice that of this study (n = 120), then the data would also have met this criteria. Table S5, Frequencies of activities presented as mean counts (SE) events per training session. F-statistic and p value from one way ANOVA. Group differences identified by post-hoc Tukey t-test; a and b indicate that there are significant differences between groups. Where data did not conform to requirements of parametric analysis, a Kruskall-Wallis test was applied followed by Mann-Whitney test to identify group differences. These measures are marked with an asterisk*. To correct for Type I errors due to multiple comparisons, the False Discovery Rate control (Benjamini & Ho [file pone.0102722.s002.doc]

Supporting Information 2. Data Tables

Table S3 Treatment Groups in Main Study.

| Treatment | A: E-Collar Group | B: Control Group 1 | C: Control Group 2 |
| --- | --- | --- | --- |
| Description | Dogs trained with remote electronic training aids by ECMA sourced trainers. | Dogs trained without the use of the e-collar by ECMA sourced trainers. | Dogs trained without the use of the e-collar by trainers by APDT members |
| **Breeds (UKKC)**  Gundog  Hound  Pastoral  Terrier  Working  Toy  Utility  Cross-Breed | 6  1  4  2  3  0  0  5 | 4  4  4  3  2  0  0  4 | 6  1  3  3  1  0  0  7 |
| **Gender**  Female Entire  Female Neuter  Male Entire  Male Neuter | 3  10  3  5 | 5  4  5  7 | 5  7  2  7 |
| **Age in months**  Mean ± SE  Range | 46.2±5.9  12 to 106 | 47.7 ± 5.8  12 to 120 | 45.1 ± 7.5  9 to 131 |
| **Reason for Referral**  Chasing  Poor Recall  Aggression | 18  1  2 | 17  4  0 | 16  4  1 |
| **Owner Estimate of Severity**  Always  Frequent  Occasional  Rare | 10  10  1  0 | 7  10  2  2 | 14  4  3  0 |

Table S4. Mean (SE) percentage of scans in posture/activity, panting, behavioural state, tail movement and position.

| Activity | GroupA | GroupB | GroupC | F_2,60_ | p | q |
| --- | --- | --- | --- | --- | --- | --- |
| Posture/Activity |  |  |  |  |  |  |
| Down | 1.45 (0.33) | 3.55 (1.09) | 3.97 (1.02) | 2.08* | 0.353 | 0.431 |
| **Sit** | **25.9 (2.64)a** | **23.6 (1.80)a** | **14.4 (1.84)b** | **8.21** | 0.0007 | **0.003** |
| **Stand** | **11.7 (1.20)a** | **16.2 (1.62)a** | **52.1 (2.32)b** | **144** | **<0.0001** | **<0.0001** |
| **Walk** | **55.0 (2.92)a** | **48.1 (1.28)a** | **23.4 (2.06)b** | **57.7** | **<0.0001** | **<0.0001** |
| Run | 1.87 (0.39) | 2.49 (0.52) | 4.64 (1.34) | 0.93* | 0.628 | 0.531 |
| State |  |  |  |  |  |  |
| Ambiguous | 8.30 (2.93) | 15.7 (5.31) | 11.9 (5.05) | 1.18* | 0.555 | 0.509 |
| *Anticipatory* | *20.8 (3.63)* | *25.5 (4.97)* | *39.5 (7.31)* | *3.16* | *0.049* | *0.092* |
| Excited | 6.05 (2.13) | 3.78 (0.84) | 7.69 (1.77) | 2.15* | 0.342 | 0.431 |
| Relaxed | 32.2 (6.37) | 29.6 (5.83) | 33.0 (7.15) | 0.09 | 0.914 | 0.67 |
| **Tense** | **24.6 (5.43)a** | **16.3 (5.25)** | **3.96 (2.06)b** | **4.99** | 0.010 | **0.027** |
| Tail Movement and Position |  |  |  |  |  |  |
| Wag | 34.1 (5.28) | 36.3 (6.07) | 41.7 (6.73) | 0.68 | 0.510 | 0.509 |
| High | 13.5 (4.53) | 15.9 (5.26) | 16.9 (6.54) | 0.11 | 0.896 | 0.67 |
| Neutral | 72.2 (6.49) | 78.6 (5.57) | 82.3 (6.47) | 0.69 | 0.505 | 0.509 |
| *Low* | *9.42 (4.42)a* | *5.87 (2.24)a* | *1.12 (0.58)b* | *5.99** | *0.050* | *0.092* |
| Panting |  |  |  |  |  |  |
| *Panting* | *20.3 (5.60)* | *9.42 (3.04)* | *12.5 (2.34)* | *3.77** | *0.152* | *0.239* |

Table S5. Frequencies of activities presented as mean counts (SE) events per training session

| Activity | Group A | Group B | Group C | F_2,60_ | p | q |
| --- | --- | --- | --- | --- | --- | --- |
| Operator Related |  |  |  |  |  |  |
| Attention seeking | 1.97 (0.95) | 3.77 (1.19) | 3.13 (0.69) | 0.88 | 0.418 | 0.324 |
| *Move Away* | *3.38 (1.04)* | *3.53 (1.01)* | *1.03 (0.49)* | *6.61** | *0.037* | 0.078 |
| **Command** | **58.8 (7.82)a** | **56.3 (6.32)a** | **32.2 (4.85)b** | **5.19** | | | **0.008** | | --- | | | --- | --- | | **0.022** |
| Lip Lick |  |  |  |  |  |  |
| **Food** | **6.13 (1.26)a** | **10.7 (2.24)b** | **20.4 (2.38)c** | **12.9** | **<0.0001** | **<0.0001** |
| *No Food* | *25.5 (4.19)* | *21.6 (4.15)* | *14.1 (1.74)* | *2.67* | *0.077* | *0.091* |
| Vocalisation |  |  |  |  |  |  |
| Bark | 0.22 (0.10) | 0.36 (0.22) | 1.00 (0.42) | 2.75* | 0.253 | 0.226 |
| *Whine* | *0.42 (0.26)* | *0.75 (0.44)* | *1.92 (0.82)* | *5.53** | *0.063* | *0.090* |
| *Yelp* | *0.55 (0.35)* | *0.10 (0.06)* | *0.14 (0.13)* | *5.28** | *0.072* | *0.090* |
| Other Events |  |  |  |  |  |  |
| Paw Lift | 7.43 (2.51) | 13.3 (4.26) | 7.04 (2.29) | 1.17 | 0.318 | 0.304 |
| **Yawn** | **0.90 (0.21)a** | **0.49 (0.13)** | **0.19 (0.06)b** | **11.1*** | **0.004** | **0.017** |
| Scratch | 0.15 (0.07) | 0.18 (0.10) | 0.68 (0.49) | 1.24* | 0.357 | 0.309 |
| Shake | 1.10 (0.22) | 1.33 (0.42) | 1.49 (0.36) | 1.92* | 0.383 | 0.309 |
| **Sniff** | **12.1 (2.00)a** | **14.3 (1.99)a** | **22.1 (2.52)b** | **5.84** | 0.005 | **0.017** |
| *Eliminate* | *0.27 (0.13)* | *0.15 (0.05)* | *0.48 (0.17)* | *5.55** | *0.062* | *0.090* |
